# Supplementary material for: Analysis of PPARγ Signaling Activity in Psoriasis
Source: Int J Mol Sci. 2021 Aug 10;22(16):8603. doi: 10.3390/ijms22168603 (PMC8395241; doi:10.3390/ijms22168603)
Supplement: Supplementary file 1 [file ijms-22-08603-s001.zip › Supplemental materials_Analysis of PPARg signaling activity in psoriasis/Pathway models/Models images and html files/Interleukin-17 and interleukin-22 signaling in psoriasis/7540.html]

MMP12


# Protein MMP12

|  |  |
| --- | --- |
| URN | urn:agi-llid:4321 |
| Total Entities | 0 |
| Connectivity | 3590 |
| Name | MMP12 |
| Description | matrix metallopeptidase 12 (macrophage elastase) |
| Notes | This gene encodes a member of the peptidase M10 family of matrix metalloproteinases (MMPs). Proteins in this family are involved in the breakdown of extracellular matrix in normal physiological processes, such as embryonic development, reproduction, and tissue remodeling, as well as in disease processes, such as arthritis and metastasis. The encoded preproprotein is proteolytically processed to generate the mature protease. This protease degrades soluble and insoluble elastin. This gene may play a role in aneurysm formation and mutations in this gene are associated with lung function and chronic obstructive pulmonary disease (COPD). This gene is part of a cluster of MMP genes on chromosome 11. [provided by RefSeq, Jan 2016] |
| Primary Cell Localization | Extracellular |

---

|  |  |
| --- | --- |
| Pathway | Proteins Involved in Atherosclerosis |
|  | Proteins Involved in Arterial Hypertension |
|  | IL22 Induces Keratinocyte Proliferation in Psoriasis |
|  | Proteins with Altered Expression in Psoriasis |
|  | Tubular Cell Dysfunction in Progressive Diabetic Nephropathy |
|  | Proteins Involved in Diabetic Nephropathy |
|  | Mesangial Cell Dysfunction in Diabetic Nephropathy |
|  | Proteins Involved in Ulcerative Colitis |
|  | Proteins Involved in Rheumatoid Arthritis |
|  | Alveolar Macrophages Dysfunction in COPD |
|  | Proteins Involved in Chronic Obstructive Pulmonary Disease |
|  | Proteins Involved in Pulmonary Emphysema |
|  | Mesangial Cell Dysfunction in Glomerulonephritis |
|  | Interstitial Fibroblasts in Pyelonephritis |
|  | BMP7/BMPR2/ACVR2 Expression Targets |
|  | CSF2 -> STAT Expression Targets |
|  | Integrins in Cancer Cell Motility, Invasion and Survival |
|  | Insulin -> STAT Expression Targets |
|  | Insulin -> CEBPA/CTNNB/FOXA/FOXO Expression Targets |
|  | Insulin -> MEF/MYOD Expression Targets |
|  | Insulin -> ELK/SRF/HIF1A/MYC/SREBF Expression Targets |
|  | IL13 Expression Targets |
|  | CSF1 -> STAT Expression Targets |
|  | CSF1 -> AP-1/CREB/CREBBP/MYC Expression Targets |
|  | TGFB1-TGFBR2 Expression Targets |
|  | TGFB1-TGFBR1/AP-1 Expression Targets |
|  | TGFB1-ACVRL1 Expression Targets |
|  | TNF -> AP-1 Expression Targets |
|  | CD72 -> AP-1 Expression Targets |
|  | TLR4 -> AP-1 Expression Targets |
|  | IL1B Expression Targets |
|  | WNT7A Expression Targets |
|  | pprarg neg, uknown targets, ps-positive |
|  | prarg neg,ukn expres targets, ps-positive |
|  | PPARG negative targets |
|  | pparg expr target\_regulators |
|  | Neighbors of disease exacerbation |
|  | Neighbors of tumor progression |
|  | Neighbors of cancer progression |
|  | Neighbors of inflammatory status |
|  | Neighbors of overall survival |
|  | Neighbors of disease severity |
|  | Neighbors of tumor size |
|  | Neighbors of lesion size |
|  | Neighbors of mortality |
|  | Neighbors of infarct size |
|  | Neighbors of survival time |
|  | Neighbors of functional recovery |
|  | Neighbors of insulin sensitivity |
|  | Neighbors of therapeutic efficacy |
|  | Neighbors of lethality |
|  | Neighbors of disease-free survival |
|  | Neighbors of macrophage count |
|  | Neighbors of body weight gain |
|  | Neighbors of microvessel density |
|  | Neighbors of tumor recurrence |
|  | Neighbors of neutrophil count |
|  | Neighbors of mortality rate |
|  | Neighbors of lesion area |
|  | Neighbors of tumor grade |
|  | Neighbors of vascular leakage |
|  | Neighbors of vascular density |
|  | Neighbors of functional status |
|  | Neighbors of infarct area |
|  | Neighbors of carotid intima-media thickness |
|  | Neighbors of disease exacerbation |
|  | Neighbors of tumor progression |
|  | Neighbors of cancer progression |
|  | Neighbors of inflammatory status |
|  | Neighbors of overall survival |
|  | Neighbors of disease severity |
|  | Neighbors of tumor size |
|  | Neighbors of lesion size |
|  | Neighbors of mortality |
|  | Neighbors of infarct size |
|  | Neighbors of survival time |
|  | Neighbors of functional recovery |
|  | Neighbors of insulin sensitivity |
|  | Neighbors of therapeutic efficacy |
|  | Neighbors of lethality |
|  | Neighbors of disease-free survival |
|  | Neighbors of macrophage count |
|  | Neighbors of body weight gain |
|  | Neighbors of microvessel density |
|  | Neighbors of tumor recurrence |
|  | Neighbors of neutrophil count |
|  | Neighbors of mortality rate |
|  | Neighbors of lesion area |
|  | Neighbors of tumor grade |
|  | Neighbors of vascular leakage |
|  | Neighbors of vascular density |
|  | Neighbors of functional status |
|  | Neighbors of infarct area |
|  | Neighbors of carotid intima-media thickness |
|  | Neighbors of cell development |
|  | Neighbors of immune response |
|  | Neighbors of inflammatory response |
|  | Neighbors of wound healing |
|  | Neighbors of angiogenesis |
|  | Neighbors of innate immune response |
|  | Neighbors of macrophage activation |
|  | Neighbors of cell formation |
|  | Neighbors of chemotaxis |
|  | Neighbors of dendritic cell differentiation |
|  | Neighbors of tissue remodeling |
|  | Neighbors of cell adhesion |
|  | Neighbors of endothelial cell proliferation |
|  | Neighbors of fibrogenesis |
|  | Neighbors of tumor growth |
|  | Neighbors of dendritic cell development |
|  | Neighbors of cell count |
|  | Neighbors of cell infiltration |
|  | Neighbors of SMC proliferation |
|  | Neighbors of adipogenesis |
|  | Neighbors of lipid storage |
|  | Neighbors of pregnancy |
|  | Neighbors of aging |
|  | Neighbors of cell motility |
|  | Neighbors of bone resorption |
|  | Neighbors of adipocyte differentiation |
|  | Neighbors of macrophage migration |
|  | Neighbors of microglial activation |
|  | Neighbors of neutrophil recruitment |
|  | Neighbors of regeneration |
|  | Neighbors of cell migration |
|  | Neighbors of cancer growth |
|  | Neighbors of phagocytosis |
|  | Neighbors of leukocyte migration |
|  | Neighbors of leukocyte recruitment |
|  | Neighbors of monocyte recruitment |
|  | Neighbors of cell invasion |
|  | Neighbors of hemopoiesis |
|  | Neighbors of lung development |
|  | Neighbors of transcription activation |
|  | Neighbors of senescence |
|  | Neighbors of hepatocyte proliferation |
|  | Neighbors of neuroprotection |
|  | Neighbors of neuronal death |
|  | Neighbors of cell growth |
|  | Neighbors of ROS generation |
|  | Neighbors of first trimester pregnancy |
|  | Neighbors of cancer cell growth |
|  | Neighbors of hemato-encephalic barrier |
|  | Neighbors of macrophage function |
|  | Neighbors of cell differentiation |
|  | Neighbors of inflammatory disease |
|  | Neighbors of rheumatoid arthritis |
|  | Neighbors of atherosclerosis |
|  | Neighbors of fibrosis |
|  | Neighbors of arthritis |
|  | Neighbors of inflammatory bowel disease |
|  | Neighbors of injury |
|  | Neighbors of atherogenesis |
|  | Neighbors of vascular remodeling |
|  | Neighbors of metastasis |
|  | Neighbors of carcinogenesis |
|  | Neighbors of multiple sclerosis |
|  | Neighbors of chronic inflammation |
|  | Neighbors of inflammation |
|  | Neighbors of pathological angiogenesis |
|  | Neighbors of obesity |
|  | Neighbors of vascular disease |
|  | Neighbors of asthma |
|  | Neighbors of pneumonia |
|  | Neighbors of osteoarthritis |
|  | Neighbors of liver fibrosis |
|  | Neighbors of chronic obstructive pulmonary disease |
|  | Neighbors of myocardial infarction |
|  | Neighbors of airway inflammation |
|  | Neighbors of insulin resistance |
|  | Neighbors of infection |
|  | Neighbors of vascular injury |
|  | Neighbors of macrophage infiltration |
|  | Neighbors of neuroinflammation |
|  | Neighbors of death |
|  | Neighbors of type 1 diabetes |
|  | Neighbors of encephalomyelitis |
|  | Neighbors of ischemic stroke |
|  | Neighbors of dermatitis |
|  | Neighbors of neutrophil infiltration |
|  | Neighbors of heart disease |
|  | Neighbors of diabetic nephropathy |
|  | Neighbors of lung injury |
|  | Neighbors of systemic sclerosis |
|  | Neighbors of hepatocellular carcinoma |
|  | Neighbors of acute lung injury |
|  | Neighbors of atherosclerotic plaque |
|  | Neighbors of experimental autoimmune encephalomyelitis |
|  | Neighbors of colorectal cancer |
|  | Neighbors of cardiac remodeling |
|  | Neighbors of breast cancer |
|  | Neighbors of melanoma |
|  | Neighbors of cartilage degeneration |
|  | Neighbors of neoplasm invasion |
|  | Neighbors of lung disease |
|  | Neighbors of pulmonary fibrosis |
|  | Neighbors of cardiovascular disease |
|  | Neighbors of stroke |
|  | Neighbors of cancer |
|  | Neighbors of cerebral ischemia |
|  | Neighbors of neoplasm |
|  | Neighbors of respiratory hypersensitivity |
|  | Neighbors of myocardial fibrosis |
|  | Neighbors of coronary artery disease |
|  | Neighbors of rupture |
|  | Neighbors of hypertension |
|  | Neighbors of epithelial cell |
|  | Neighbors of vascular smooth muscle cell |
|  | Neighbors of monocyte |
|  | Neighbors of alveolar macrophage |
|  | Neighbors of macrophage |
|  | Neighbors of activated monocyte |
|  | Neighbors of fibroblast |
|  | Neighbors of airway epithelial cell |
|  | Neighbors of stromal cell |
|  | Neighbors of airway smooth muscle cell |
|  | Neighbors of microglia |
|  | Neighbors of trophoblast |
|  | Neighbors of mononuclear cell |
|  | Neighbors of dendritic cell |
|  | Neighbors of inflammatory cell |
|  | Neighbors of bone marrow macrophage |
|  | Neighbors of peritoneal macrophage |
|  | Neighbors of osteoclast |
|  | Neighbors of neutrophil |
|  | Neighbors of smooth muscle myocyte |
|  | Neighbors of astrocyte |
|  | Neighbors of activated T-cell |
|  | Neighbors of granulocyte |
|  | Neighbors of myeloid cell |
|  | Neighbors of cytotrophoblast |
|  | Neighbors of tumor infiltrating macrophage |
|  | Neighbors of B-cell |
|  | Neighbors of T-cell |
|  | Neighbors of M1 macrophage |
|  | Neighbors of myofibroblast |
|  | Neighbors of adipose macrophage |
|  | Neighbors of brain cell |
|  | Neighbors of M2 macrophage |
|  | Neighbors of lung |
|  | Neighbors of skin |
|  | Neighbors of airway |
|  | Neighbors of blood vessel |
|  | Neighbors of aorta |
|  | Neighbors of esophagus |
|  | Neighbors of artery |
|  | Neighbors of cardiovascular system |
|  | Neighbors of coronary artery |
|  | Neighbors of vitreous body |
|  | Neighbors of NF-kB family |
|  | Neighbors of JNK |
|  | Neighbors of IL1 family |
|  | Neighbors of PPAR |
|  | Neighbors of Jun/Fos |
|  | Neighbors of NFKBI |
|  | Neighbors of PI3K |
|  | Neighbors of STAT family |
|  | Neighbors of mitogen-activated protein kinase |
|  | Neighbors of transforming growth factor |
|  | Neighbors of cytokine |
|  | Neighbors of MEK1/2 |
|  | Neighbors of PDGF |
|  | Neighbors of ERK1/2 |
|  | Neighbors of growth factor |
|  | Neighbors of PKA |
|  | Neighbors of ovalbumin |
|  | Neighbors of LDL |
|  | Neighbors of NAD(P)H oxidase |
|  | Neighbors of SMAD subfamily |
|  | Neighbors of inflammatory cytokine |
|  | Neighbors of PDGF-BB |
|  | Neighbors of GPCR |
|  | Neighbors of proteasome endopeptidase complex |
|  | Neighbors of STAT5 |
|  | Neighbors of WNT |
|  | Neighbors of gonadotropins |
|  | Neighbors of ITG |
|  | Neighbors of pancreatic elastase |
|  | PPARG negative regulators and targets |
|  | Model of PPARG related pathways in psoriasis (short version) |
|  | 2\_Interleukin-17 and interleukin-22 signaling in psoriasis |
|  | 3\_The role of interstitial fibroblasts in pyelonephritis |
|  | 3\_Mesangial cell dysfunction in glomerulonephritis |
|  | 3\_Role of alveolar macrophages in pulmonary tissue destruction in COPD |
|  | Interleukin-17 and interleukin-22 signaling in psoriasis |

---

|  |  |
| --- | --- |
| MedScan ID | 4321 |

---

|  |  |
| --- | --- |
| LocusLink ID | 4321 |
|  | 17381 |
|  | 117033 |

---

|  |  |
| --- | --- |
| Alias | matrix metallopeptidase 12 |
|  | matrix metallopeptidase 12 (macrophage elastase) |
|  | Macrophage metalloelastase |
|  | human macrophage metalloelastase |
|  | matrix metalloprotease 12 (macrophage elastase) |
|  | metalloelastase |
|  | matrix metalloprotease 12 |
|  | macrophage elastase |
|  | MGC138506 |
|  | MMP-12 |
|  | matrix metalloproteinase 12 (macrophage elastase) |
|  | AV378681 |
|  | Matrix metalloproteinase XII |
|  | Matrix metalloprotease XII |
|  | Mmel |
|  | matrix metallopeptidase XII |
|  | human macrophage metalloelastases |
|  | metalloelastases |
|  | OTTMUSP00000034110 |
|  | matrix metalloproteinase 12 |
|  | ME |
|  | HME |
|  | MME |
|  | matrix metalloproteinase-12 |
|  | macrophage-metalloelastase |
|  | Mmp12 |

---

|  |  |
| --- | --- |
| EC Number | 3.4.24.65 |

---

|  |  |
| --- | --- |
| GO ID | 0005509 |
|  | 0005518 |
|  | 0001047 |
|  | 0004175 |
|  | 0004222 |
|  | 0043565 |
|  | 0008270 |
|  | 0098586 |
|  | 0030574 |
|  | 0060309 |
|  | 0022617 |
|  | 0030198 |
|  | 1904905 |
|  | 0000122 |
|  | 0060339 |
|  | 0060054 |
|  | 1902741 |
|  | 0045944 |
|  | 0060340 |
|  | 0006606 |
|  | 0006508 |
|  | 0050691 |
|  | 1904645 |
|  | 0035313 |
|  | 0005737 |
|  | 0031012 |
|  | 0005576 |
|  | 0005615 |
|  | 0005634 |
|  | 0016787 |
|  | 0046872 |
|  | 0008237 |
|  | 0008233 |
|  | 0010628 |
|  | 1901163 |
|  | 0042060 |
|  | 0050679 |
|  | 0042493 |
|  | 0001666 |
|  | 0014070 |
|  | 0004252 |
|  | 0005578 |
|  | 0008152 |
|  | 0042221 |

---

|  |  |
| --- | --- |
| KEGG ID | hsa:4321 |
|  | mmu:17381 |
|  | rno:117033 |

---

|  |  |
| --- | --- |
| Organism | Homo sapiens {Organism urn:agi-taxid:9606} |
|  | Mus musculus {Organism urn:agi-taxid:10090} |
|  | Rattus norvegicus {Organism urn:agi-taxid:10116} |
|  | Homo sapiens |
|  | Mus musculus |
|  | Rattus norvegicus |

---

|  |  |
| --- | --- |
| Mouse chromosome position | 9 2.46 cM |
|  | 9 1.0 cM |

---

|  |  |
| --- | --- |
| OMIM ID | 601046 |

---

|  |  |
| --- | --- |
| Rat chromosome position | 8q11 |

---

|  |  |
| --- | --- |
| Hugo ID | 7158 |
|  | HGNC:7158 |

---

|  |  |
| --- | --- |
| Human chromosome position | 11q22.2 |
|  | 11q22.3 |

---

|  |  |
| --- | --- |
| Swiss-Prot Accession | P39900 |
|  | P39900.1 |
|  | P34960 |
|  | Q3TCW6 |
|  | P34960.3 |
|  | Q63341 |
|  | R9PXT7 |
|  | Q63341.1 |
|  | B2R9X8 |
|  | B7ZLF6 |
|  | Q2M1L9 |
|  | Q3TB28 |
|  | Q3TBV6 |
|  | Q3TBV9 |
|  | Q3TD61 |
|  | Q3U1S8 |
|  | Q3U1S9 |
|  | Q3U2R8 |
|  | Q3UBC5 |
|  | Q4FJM7 |
|  | Q8BJ92 |
|  | Q8BJB3 |
|  | Q8BJB6 |
|  | Q8BJB8 |
|  | Q8BJB9 |
|  | Q8VED6 |
|  | Q5I0P0 |
|  | Q13165 |
|  | Q99745 |
|  | Q6GX99 |
|  | Q8BJC0 |

---

|  |  |
| --- | --- |
| PIR ID | A49499 |
|  | A42401 |

---

|  |  |
| --- | --- |
| GenBank ID | NC\_000011 |
|  | NM\_002426 |
|  | NP\_002417 |
|  | NG\_032936 |
|  | AP000789 |
|  | AY856072 |
|  | AAW29944 |
|  | CH471065 |
|  | EAW67033 |
|  | FP885831 |
|  | U25346 |
|  | AAB06314 |
|  | U78045 |
|  | AAB36943 |
|  | AI343504 |
|  | AI352562 |
|  | AK313959 |
|  | BAG36675 |
|  | AW172994 |
|  | BC112301 |
|  | AAI12302 |
|  | BC143773 |
|  | AAI43774 |
|  | BE440047 |
|  | BE813419 |
|  | BG206655 |
|  | L23808 |
|  | AAA58658 |
|  | P39900 |
|  | NC\_000075 |
|  | NM\_001320077 |
|  | NP\_001307006 |
|  | XM\_017313160 |
|  | XP\_017168649 |
|  | NM\_001320076 |
|  | NP\_001307005 |
|  | NM\_008605 |
|  | NP\_032631 |
|  | AC169512 |
|  | CH466522 |
|  | EDL24933 |
|  | AK089295 |
|  | BAC40832 |
|  | AK089309 |
|  | BAC40836 |
|  | AK089335 |
|  | BAC40846 |
|  | AK089452 |
|  | BAC40889 |
|  | AK089509 |
|  | BAC40909 |
|  | AK089523 |
|  | BAC40913 |
|  | AK090051 |
|  | BAC41067 |
|  | AK150596 |
|  | BAE29689 |
|  | AK151021 |
|  | BAE30039 |
|  | AK151273 |
|  | BAE30260 |
|  | AK151933 |
|  | BAE30809 |
|  | AK155139 |
|  | BAE33072 |
|  | AK155743 |
|  | BAE33411 |
|  | AK155747 |
|  | BAE33414 |
|  | AK155748 |
|  | BAE33415 |
|  | AK155841 |
|  | AK155865 |
|  | BAE33469 |
|  | AK170360 |
|  | BAE41743 |
|  | AK170364 |
|  | BAE41746 |
|  | AK170500 |
|  | BAE41839 |
|  | AK170667 |
|  | AK170798 |
|  | BAE42035 |
|  | AK170804 |
|  | BAE42039 |
|  | AK170976 |
|  | BAE42154 |
|  | AK171006 |
|  | BAE42177 |
|  | AK171032 |
|  | BAE42198 |
|  | AK171036 |
|  | BAE42201 |
|  | AK171176 |
|  | BAE42294 |
|  | AK171428 |
|  | BAE42446 |
|  | AK171435 |
|  | AK171447 |
|  | BAE42457 |
|  | AK171448 |
|  | BAE42458 |
|  | AK171449 |
|  | BAE42459 |
|  | AK171450 |
|  | BAE42460 |
|  | AK171466 |
|  | BAE42472 |
|  | AK171468 |
|  | BAE42474 |
|  | AK171470 |
|  | BAE42476 |
|  | AK171472 |
|  | BAE42478 |
|  | AK171485 |
|  | BAE42486 |
|  | AK171518 |
|  | BAE42502 |
|  | AK171542 |
|  | BAE42516 |
|  | AK171570 |
|  | BAE42531 |
|  | AY622972 |
|  | AAT46403 |
|  | BC019135 |
|  | AAH19135 |
|  | BY195619 |
|  | BY539785 |
|  | CT010377 |
|  | CAJ18584 |
|  | M82831 |
|  | AAA39526 |
|  | P34960 |
|  | NC\_005107 |
|  | XM\_017595420 |
|  | XP\_017450909 |
|  | NM\_053963 |
|  | NP\_446415 |
|  | AC\_000076 |
|  | AAHX01053075 |
|  | AC120947 |
|  | CH473993 |
|  | EDL78537 |
|  | BC088120 |
|  | AAH88120 |
|  | CB791502 |
|  | X98517 |
|  | CAA67142 |
|  | Q63341 |
|  | NC\_018922 |
|  | AMYH02025489 |
|  | HQ258263 |
|  | ADR83017 |
|  | XM\_011242358 |
|  | XP\_011240660 |
|  | XM\_011242357 |
|  | XP\_011240659 |
|  | AC\_000031 |
|  | AAHY01075863 |
|  | AC\_000143 |
|  | ABBA01039930 |
|  | XM\_006509847 |
|  | XP\_006509910 |
|  | NW\_003871077 |
|  | NT\_033899 |
|  | NW\_001838042 |
|  | NW\_925173 |
|  | AC\_000054 |
|  | CR594246 |
|  | CR603756 |
|  | Q13165 |
|  | Q99745 |
|  | NT\_039471 |
|  | NW\_001030907 |
|  | Q3TCW6 |
|  | Q6GX99 |
|  | Q8BJC0 |
|  | NW\_001084867 |
|  | NW\_047798 |

---

|  |  |
| --- | --- |
| Swiss-Prot ID | MMP12\_HUMAN |
|  | MMP12\_MOUSE |
|  | MMP12\_RAT |
|  | B2R9X8\_HUMAN |

---

|  |  |
| --- | --- |
| Cell Localization | Secreted |
|  | Extracellular space |
|  | Extracellular matrix |
|  | Secreted, extracellular space, extracellular matrix |
|  | Extracellular |

---

|  |  |
| --- | --- |
| Ensembl ID | ENSG00000262406 |
|  | ENSP00000458585.1 |
|  | ENST00000571244.2 |
|  | ENSMUSG00000049723 |
|  | ENSMUSP00000114129.1 |
|  | ENSMUST00000120655.7 |
|  | ENSMUSP00000005950.5 |
|  | ENSMUST00000005950.11 |
|  | ENSRNOG00000030187 |
|  | ENSRNOP00000011727.6 |
|  | ENSRNOT00000011727.6 |
|  | ENSP00000458585 |
|  | ENST00000571244 |
|  | ENSMUSP00000005950 |
|  | ENSMUST00000005950 |
|  | ENSMUSP00000114129 |
|  | ENSMUST00000120655 |
|  | ENSRNOP00000011727 |
|  | ENSRNOT00000011727 |

---

|  |  |
| --- | --- |
| MGI ID | MGI:97005 |
|  | 97005 |

---

|  |  |
| --- | --- |
| RGD ID | 620195 |

---

|  |  |
| --- | --- |
| Unigene ID | Hs.1695 |
|  | Hs.709832 |
|  | Mm.2055 |
|  | Rn.33193 |

---

|  |  |
| --- | --- |
| Homologene ID | 20547 |

---

|  |  |
| --- | --- |
| IPI ID | IPI00298246 |
|  | IPI00475653 |
|  | IPI00321648 |
|  | IPI00881760 |
|  | IPI00881624 |
|  | IPI00403307 |
|  | IPI00882065 |
|  | IPI00199747 |

---
